# CircRNA: Functions, Applications and Prospects

**DOI:** 10.3390/biom14121503

**Published:** 2024-11-26

**Authors:** Fei Xu, Qing Xiao, William W. Du, Sheng Wang, Burton B. Yang

**Affiliations:** 1 Sunnybrook Research Institute, Sunnybrook Health Sciences Centre, Toronto, ON M4N 3M5, Canada; xufei891221@163.com (F.X.); xiaoqingxiaomeng@163.com (Q.X.); 2 Department of Anesthesiology, Beijing Anzhen Hospital, Capital Medical University, Beijing 100029, China; shengwang_gz@163.com; 3 Department of Laboratory Medicine and Pathobiology, University of Toronto, Toronto, ON M5S 1A8, Canada

## 1. Functions of Circular RNAs

Circular RNAs (circRNAs) represent a distinct class of covalently closed non-coding RNA molecules characterized by the absence of free 5′ and 3′ ends. The first circRNAs were identified in plant viroids, where their existence was subsequently confirmed in eukaryotic cells through electron microscopy [1]. In recent years, advancements in high-throughput research methodologies, bioinformatics technologies, and molecular bioassays have facilitated a deeper understanding of the function of circRNAs, revealing their ubiquitous expression and developmental roles. Despite the identification of over one million circRNAs to date, only a small subset has been functionally characterized. Notably, circRNAs have been found to exhibit cell-specific and tissue-specific spatial expression patterns across various cell types and tissues [2,3]. The dysregulation of circRNAs has been observed in the development and pathogenesis involved in diverse diseases, including cancers, central nervous system disorders, and diseases in the heart and lung.

This Special Issue entitled “Circular RNAs: Functions, Applications and Prospects” of *biomolecules* comprises a total of seven contributions, including five reviews and two original articles, to provide new insight into circular RNAs.

Sanadgol et al. [4] detected the interaction of circPSEN1s with eight specific microRNAs and nine proteins impacting TGF-β and Notch signaling pathways, which are associated with the progression of Alzheimer’s disease (AD). Xuan et al. [5] discovered the temporal specificity of circRNA expression in mammary gland tissues across various developmental stages. This study established the competitive endogenous RNA (ceRNA) regulatory networks of circRNA–miRNA–mRNA that are pertinent to mammary development, immune response, metabolic processes, and apoptosis.

The review titled “Circular RNA Expression Profiling by Microarray—A Technical and Practical Perspective” by Yanggu Shi and Shangjin Dong [6] summarizes the application of circRNA microarrays as a practical and effective technology for circRNA profiling, suitable for use in well-equipped biological or clinical research laboratories. “The Essentials on microRNA-Encoded Peptides from Plants to Animals” [7] presents the latest advancements in the research of microRNA-encoded peptides (miPEPs), highlighting their potential applications as valuable in disease treatment.

Tan et al. [8] elucidate the role of non-coding RNAs in modulating neural stem cells (NSCs) within the hippocampus and explore their potential applications in treatment. The review entitled “Role of circRNA in E3 Modification under Human Disease” [9] presents the characteristics of circRNA in ubiquitination, summarizing the mechanisms by which circRNAs regulate ubiquitination across various diseases. “Circular RNAs and Their Role in Male Infertility: A Systematic Review” [10] summarizes the differential expression of circRNAs in male infertility and identifies circRNAs as candidate biomarkers.

The majority of circRNAs are produced through the back-splicing of precursor mRNA by RNA polymerase II. This process involves the joining of the 5′ splice site (splice donor) to an upstream 3′ splice site (splice acceptor), resulting in the formation of a circular RNA molecule characterized by a 3′–5′ phosphodiester bond at the back-splicing junction site (BSJ) [11]. CircRNAs can be classified into three categories based on their genomic origin and cycling mechanism: exonic circRNAs (ecircRNAs), intronic circRNAs (ciRNAs), and exon-intron circRNAs (EIciRNAs) [12]. Although originally generated in the nucleus, circRNAs are predominantly detected in the cytoplasm. Most ecircRNAs are transported to the cytoplasm via the nuclear export system, while the majority of ciRNAs and EIciRNAs are retained in the nucleus.

CircRNAs play a significant role in various aspects of gene expression, influencing critical processes from transcription in the nucleus to translation in the cytoplasm [13]. Several mechanisms have been proposed regarding the impact of circRNAs on RNA polymerase II (RNA Pol II) transcription. Specifically, circRNAs can form R-loops with their corresponding genomic loci, thereby affecting transcription. Additionally, circRNAs may coactivate transcription factors (TFs), further modulating transcriptional activity. CircRNAs are generated from the middle exons of protein-coding genes, which means that the utilization of 5′ and 3′ splice sites during circRNA back-splicing can compete with the splicing of pre-mRNA [14]. In the cytoplasm, circRNAs may function as microRNA sponges, modulating the accessibility of microRNAs to their target mRNAs. Additionally, circRNAs can directly bind to mRNAs, thereby influencing gene expression outcomes. Furthermore, circRNAs can also interact with circRNA-binding proteins (cRBPs), modulating their function, distribution, and interaction with other proteins [15]. CircRNAs can also compete with linear mRNAs by binding to the same proteins, which can lead to alterations in mRNA translation. Emerging evidence indicates that certain circRNAs contain open reading frames (ORFs), internal ribosome entry site (IRES) elements, and N6-methyladenosine-modified nucleotide sequences, and are associated with cap-independent translation initiation factors and polyribosomes. Since 2017, an increasing number of circRNAs has been reported to encode functional proteins [16], highlighting the roles of circRNAs in gene expression via translation.

## 2. Applications and Perspective

CircRNAs are recognized for their resistance to exonuclease-mediated degradation, which contribute to their specificity and stability. Furthermore, they often exhibit tissue-specific and developmental stage-specific expression that is independent of their corresponding linear RNA counterparts [17]. Given these advantages, research in this field has expanded beyond functional and mechanistic studies to include the potential applications of circRNAs in the clinical treatment of human diseases. Advances in RNA sequencing and bioinformatics technologies have revealed the widespread presence of circRNAs in eukaryotic transcriptomes. Consequently, these molecules have emerged as promising biomarkers for the diagnosis and gene therapy of human diseases, making them a significant focus of contemporary research.

The expression and regulatory mechanisms of circRNAs are often closely associated with the expression of their host genes and the development of various diseases. Consequently, it is feasible to either knock out or overexpress specific circRNAs using genome-editing tools to attain therapeutic outcomes. The overexpression of circRNAs can be achieved through the application of plasmids and viral vectors, while silencing can be accomplished using small interfering RNAs (siRNAs) or deoxyribozymes (DNAzymes). Lipid nanoparticles (LNPs) are currently the most widely utilized nanocarriers for RNA delivery, including circRNAs [18]. Upon endocytosis, lipid nanoparticles (LNPs) destabilize the endosomal membrane, facilitating the release of circRNAs into the cytoplasm. Lentiviral, adenoviral or adeno-associated virus (AAV)-expressing vectors represent effective methodologies for the in vivo delivery and overexpression of circRNAs.

Exosomes derived from diverse sources serve as effective delivery vehicles for the transport and modulation of RNA levels. A notable advantage of exosome-mediated delivery is that exosomes safeguard RNAs from degradation and facilitate cellular uptake without eliciting immune responses [19]. Furthermore, circRNAs contained within exosomes represent a significant class of biomarkers, which are abundantly present in plasma. These circRNAs are considered instrumental in advancing drug development, thereby offering additional avenues for future in vivo research.

Certain circRNA-encoded proteins or peptides are involved in various signaling pathways and serve as significant regulators in disease processes by affecting cell proliferation, migration, apoptosis, and other critical biological functions [20]. Consequently, circRNA-encoded proteins or peptides present considerable potential as therapeutic targets for pharmacological interventions. The development of precision drugs targeting proteins or peptides translated from circRNAs may result in highly selective and minimally toxic therapeutic outcomes.

A comparative analysis of circRNA datasets from humans and mice revealed that 15% of circRNAs correspond to conserved splice sites in orthologous genes. In heart tissues, up to 10% of circRNAs are evolutionarily conserved across humans, mice, and rats. This conservation enhances the potential for translational research, allowing for the application of findings from animal models to clinical research [21].

The third-generation sequencing technology, utilizing nanopore sequencing, holds significant promise for the comprehensive full-length profiling of circRNAs within human transcriptomes, facilitating a deeper understanding of circRNA biology. It is anticipated that through the effective validation of sequencing results and thorough investigations into circRNA expression and regulation, a greater number of functional circRNAs will be identified as potential biomarkers and therapeutic targets, which may serve as therapeutic agents in the future.
